# Deciphering the role of cuproptosis‐related lncRNAs in shaping the lung cancer immune microenvironment: A comprehensive prognostic model

**DOI:** 10.1111/jcmm.18519

**Published:** 2024-07-08

**Authors:** Hai Huang, Guoxi Chen, Zongqi Zhang, Gang Wu, Zhengbin Zhang, Aiping Yu, Jianjie Wang, Chao Quan, Yuehua Li, Meilan Zhou

**Affiliations:** ^1^ Tuberculosis ward No.2, Wuhan Pulmonary Hospital Wuhan Institute for Tuberculosis Control Wuhan Hubei China; ^2^ Department of Tuberculosis control, Wuhan Pulmonary Hospital Wuhan Institute for Tuberculosis Control, Affiliated to Jianghan University Wuhan Hubei China; ^3^ Infectious disease prevention and control department Dongxihu Centers for Disease Prevention and Control Wuhan Hubei China; ^4^ Wuhan Pulmonary Hospital, Wuhan Institute for Tuberculosis Control Affiliated to Jianghan University Wuhan Hubei China

**Keywords:** cuproptosis, IncRNA, lung cancer

## Abstract

Cuproptosis plays an important role in cancer, but its role in lung cancer remains unknown. Transcriptional profiles, clinical details and mutation data were acquired from the Cancer Genome Atlas database through a variety of methods. The analysis of this publicly available data was comprehensively performed using R software along with its relevant packages, ensuring a thorough examination of the information. In this study, we conducted a detailed analysis of cuproptosis‐related genes and lncRNA co‐expression, identifying 129 relevant lncRNAs and establishing a prognostic model with four key lncRNAs (LINC00996, RPARP‐AS1, SND1‐IT1, TMPO‐AS1). Utilizing data from TCGA and GEO databases, the model effectively categorized patients into high‐ and low‐risk groups, showing significant survival differences. Correlation analysis highlighted specific relationships between individual lncRNAs and cuproptosis genes. Our survival analysis indicated a higher survival rate in the low‐risk group across various cohorts. Additionally, the model's predictive accuracy was confirmed through independent prognostic analysis and ROC curve evaluations. Functional enrichment analysis revealed distinct biological pathways and immune functions between risk groups. Tumour mutation load analysis differentiated high‐ and low‐risk groups by their mutation profiles. Drug sensitivity analysis and immune infiltration studies using the CIBERSORT algorithm further elucidated the potential treatment responses in different risk groups. This comprehensive evaluation underscores the significance of lncRNAs in cuproptosis and their potential as biomarkers for lung cancer prognosis and immune microenvironment.

## 1 INTRODUCTION

Lung cancer, encompassing both non‐small cell lung cancer (NSCLC) and small cell lung cancer (SCLC), stands as a principal cause of cancer‐related mortality worldwide.^1^ Despite extensive research and technological advancements in medical treatment over the years, the overall survival rate for lung cancer patients remains disappointingly low, often hampered by late‐stage diagnosis and the challenge of overcoming drug resistance.^2^ Conventional treatment approaches, including surgical resection, radiation therapy, and chemotherapy, while instrumental, can lead to significant side effects and impact the overall wellbeing of patients.^3^ Against this backdrop, the role of bioinformatics has surged to prominence. Leveraging this multidisciplinary field, researchers and clinicians are now better equipped to unravel the genetic and molecular complexities of lung cancer. This advancement not only paves the way for the identification of novel biomarkers and therapeutic targets but also fosters the development of personalized treatment plans.^4^ Consequently, this has the potential to significantly enhance early detection, treatment efficacy, and ultimately, the survival and quality of life of patients afflicted with lung cancer.

Cuproptosis, a recently delineated form of cell death, diverges fundamentally from known processes like apoptosis and necrosis, primarily driven by the perturbation of cellular copper homeostasis.^5^ This phenomenon has rapidly gained traction in cancer research, especially regarding its potential implications in the context of NSCLC.^6^ The induction of cell death via copper modulation offers a novel perspective on the intricate network of oncological pathways.^6^ Compounding this complexity, long non‐coding RNAs (lncRNAs) have been recognized as pivotal regulators in myriad cellular functions, including cuproptosis.^7^ These lncRNAs, orchestrating gene expression and molecular interactions, may significantly influence the susceptibility of NSCLC cells to copper‐mediated cytotoxicity.^8^ The convergence of cuproptosis with lncRNA‐mediated regulation heralds a new frontier in NSCLC research, hinting at unprecedented therapeutic targets and strategies. Delving into the interplay between lncRNAs and cuproptosis could shed light on the nuanced molecular mechanisms underpinning NSCLC development and resistance, thereby charting the course for ground‐breaking treatment modalities that exploit the unique vulnerabilities of cancer cells.

In our investigation, we embarked on an in‐depth exploration of the potential influence exerted by cuproptosis‐related lncRNAs in NSCLC. Our systematic approach encompassed a suite of analytical techniques, including Cox regression analysis, pathway enrichment analysis, mutational profiling, drug sensitivity analysis and immune response assessment. This comprehensive analysis culminated in the successful establishment and validation of a cuproptosis‐related lncRNA signature. These insights not only deepen our understanding of the role played by cuproptosis‐related lncRNAs in NSCLC but also pave the way for the development of tailored therapeutic strategies, enhancing individualized patient care.

## 2 METHODS

### 2.1 Data retrieval and organization

RNA sequence data and clinical characteristics of lung cancer patients from The Cancer Genome Atlas (TCGA) database (1043 NSCLC tumour samples) and gene expression omnibus (GEO) database (167 NSCLC tumour samples).^9^, ^10^ Distinguishing gene expression matrices as mRNA and lncRNA, the clinical data of lncRNA and samples were used as the basis for subsequent studies. Then we screened 19 genes related to copper apoptosis from previous literature and obtained and cuproptosis‐related lncRNAs through co‐expression analysis as a way to construct a prognostic model. The process of data processing included: raw data download, probe annotation, missing value complementation, and removal of inter‐P differences. This processing was performed by two professional bioinformatics analysts. The data were analysed using the software R4.2.2.

### 2.2 Construction of the prognostic model

We divided the NSCLC tumour samples into a training set and a test set, the training cohort set was subjected to LASSO regression analysis to obtain more representative genes, and then one‐way Cox regression was performed to screen potential prognostic genes. Genes showing significance (*p*‐value <0.05) in Cox analysis were considered potential prognostic genes. In the training cohort, patients were categorized into low‐ and high‐risk groups using the median risk score as the cut‐off point. The model formula of the training set was then validated against the samples in the validation set to obtain the risk value of each sample in the validation set and verify the accuracy of the model. In addition to further validating the reliability of the model, we used 167 NSCLC samples from the GEO database as an external test set to further verify the accuracy of the model.

### 2.3 Correlation analysis

We obtained the relationship between the modelled lncRNAs and copper death‐related genes by performing correlation analysis between the copper death‐related genes and the modelled lncRNAs to clarify the relationship between the different lncRNAs and copper death genes and to further understand the relationship between these lncRNAs and the relevant pathways of copper metabolism.

### 2.4 Survival analysis

Survival differences between high‐ and low‐risk groups were further analysed by ROC analysis to further assess the prognostic ability of the genetic traits. To further validate the accuracy of model prediction, we tested the accuracy of our model between different subgroups by grouping the samples and performing survival analysis separately for different genders and different stages.

### 2.5 Independent prognostic analysis

We performed univariate and multivariate prognostic analyses on the age, gender, staging and grading of the samples as well as on the risk values for which we constructed models, to further test whether the models we constructed could predict the prognosis of the samples independently of other factors.

### 2.6 Principal component analysis (PCA)

We performed the PCA of all genes, cuproptosis‐related genes, cuproptosis‐related lncRNAs, and lncRNAs involved in model construction by limma and scatterplot3d packages to further verify whether the model genes we constructed could differentiate samples between high and low groups.

### 2.7 Immune‐related functional analysis

Through the immune‐related function analysis, we further obtained the immune‐related functions that differed between the high‐ and low‐risk groups, which provided a reference for subsequent studies.^11^

### 2.8 Functional enrichment analysis

Combining the risk value of each sample and the gene expression matrix in the samples, we performed functional enrichment analysis on the differential genes in the high‐ and low‐risk groups. Through GO/KEGG functional enrichment analysis, based on the pathways with differential expression between the high‐ and low‐risk groups in the results, we set up the filtering conditions and then took the pathways that were more obvious in the high‐ and low‐expression groups as the objects of the next step of research.^12^

### 2.9 Analysis of differences in TMB

Through the tumour mutation load data of the samples in the TCGA database, combined with the risk values of the samples, we analysed the differences in mutation loads in the high‐ and low‐risk groups and analysed the mutation differences of the modelled genes between the high‐ and low‐risk groups, which further revealed the mechanism of tumour mutations.

### 2.10 Immune infiltration analysis

The CIBERSPRT algorithm was used to quantify the specific immune component in the lung cancer immune microenvironment.^13^

### 2.11 Tumour immune dysfunction and exclusion (TIDE)

Through the website (http://tide.dfci.harvard.edu), we analyse the analysis of tumour samples on immunotherapy escape in non‐small cell lung cancer, combining the risk values of each sample in the model, and perform a comparative analysis to compare the differences between immunotherapy escape between high‐ and low‐risk groups.^14^

### 2.12 Drug sensitivity analysis

Combined with the data files of drug sensitivity in the database, we scored the drug sensitivity of each sample and then combined with the risk value of each sample, we analysed the sensitivity of high‐ and low‐risk groups to different drugs.^15^

## 3 RESULTS

**FIGURE 1:**
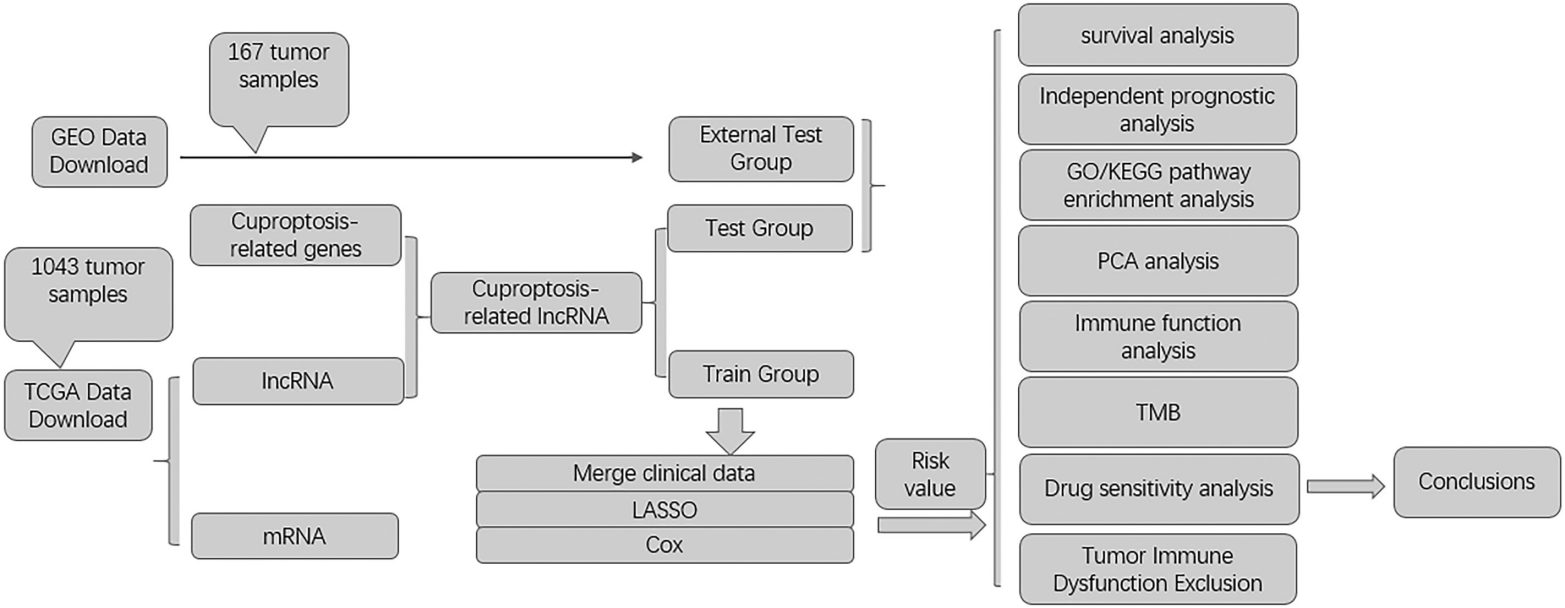
Flow chart of the entire study.

The flow chart of the study is shown in Figure 1.

### 3.1 Cuproptosis‐related genes and lncRNA co‐expression analysis results

**FIGURE 2:**
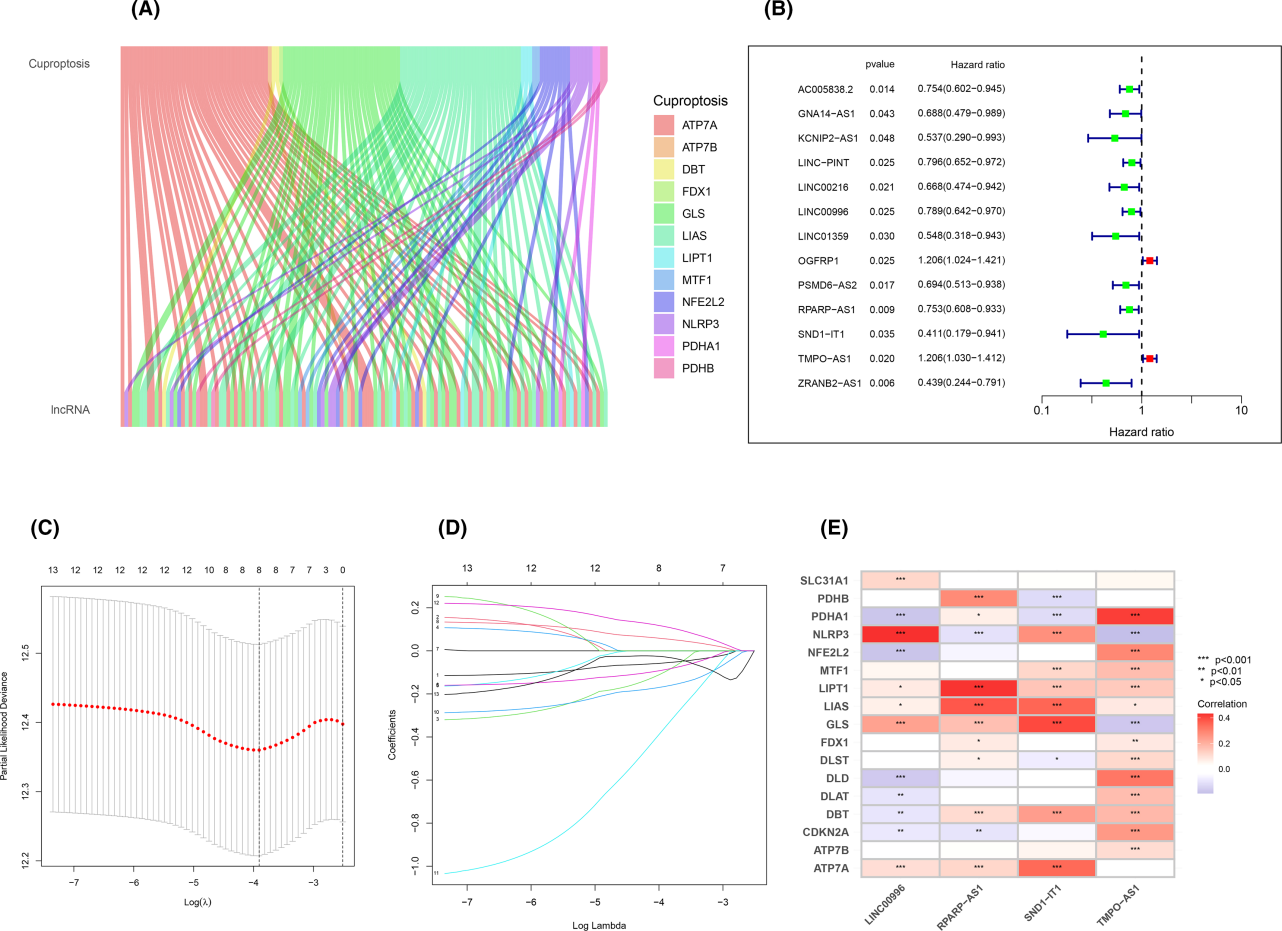
Correlation analysis results. (A) The correlation analysis results between copper death and lncRNA indicate that there are connections between different modules, and different colours represent different copper death related genes. (B) The result of Cox regression analysis, the red dot represents high risk, while the green dot represents low risk. (C, D) The result of Lasso regression analysis, It can be seen that modelling six genes is more accurate and reliable. (E) Correlation analysis between the lncRNAs in the model and the cuproptosis‐related genes, red represents positive correlation, while blue represents negative correlation.

With co‐expression analysis, we obtained 129 cuproptosis‐related lncRNAs and visualized the relationship between cuproptosis genes and lncRNAs by the Sankey diagram, and the results are displayed in Figure 2A.

### 3.2 Construction of the prognostic model

We divided the tumour samples downloaded from the TCGA database into training and validation sets, one‐way Cox regression was performed by the samples in the training cohort to screen potential prognostic genes. The results are displayed in Figure 2B, After LASSO regression analysis to get the more representative genes, and the results are displayed in Figure 2C,D. Finally, a prognostic model was obtained by constructing a prognostic model with four cuproptosis‐associated lncRNAs (LINC00996, RPARP‐AS1, SND1‐IT1, TMPO‐AS1). Based on the prognostic model the risk value of each sample was obtained, and the patients were categorized into low‐ and high‐risk groups, using the median risk score as the cut‐off point.

### 3.3 Correlation analysis between lncRNAs and cuproptosis genes

We clarified the relationship between different lncRNAs and cuproptosis genes by performing correlation analysis between cuproptosis‐related genes and the lncRNAs used to construct the model to get the relationship between the lncRNAs and cuproptosis‐related genes in the model, and it can be seen that the strongest cuproptosis‐related genes correlated with LINC00996 were NLRP3; the strongest cuproptosis‐related genes correlated with RPARP−AS1 were LIPT1; the strongest cuproptosis‐related genes correlated with SND1‐IT1 were GLS; the strongest cuproptosis‐related genes correlated with TMPO‐AS1were PDHA1, the results are displayed in Figure 2E.

### 3.4 Survival analysis

**FIGURE 3:**
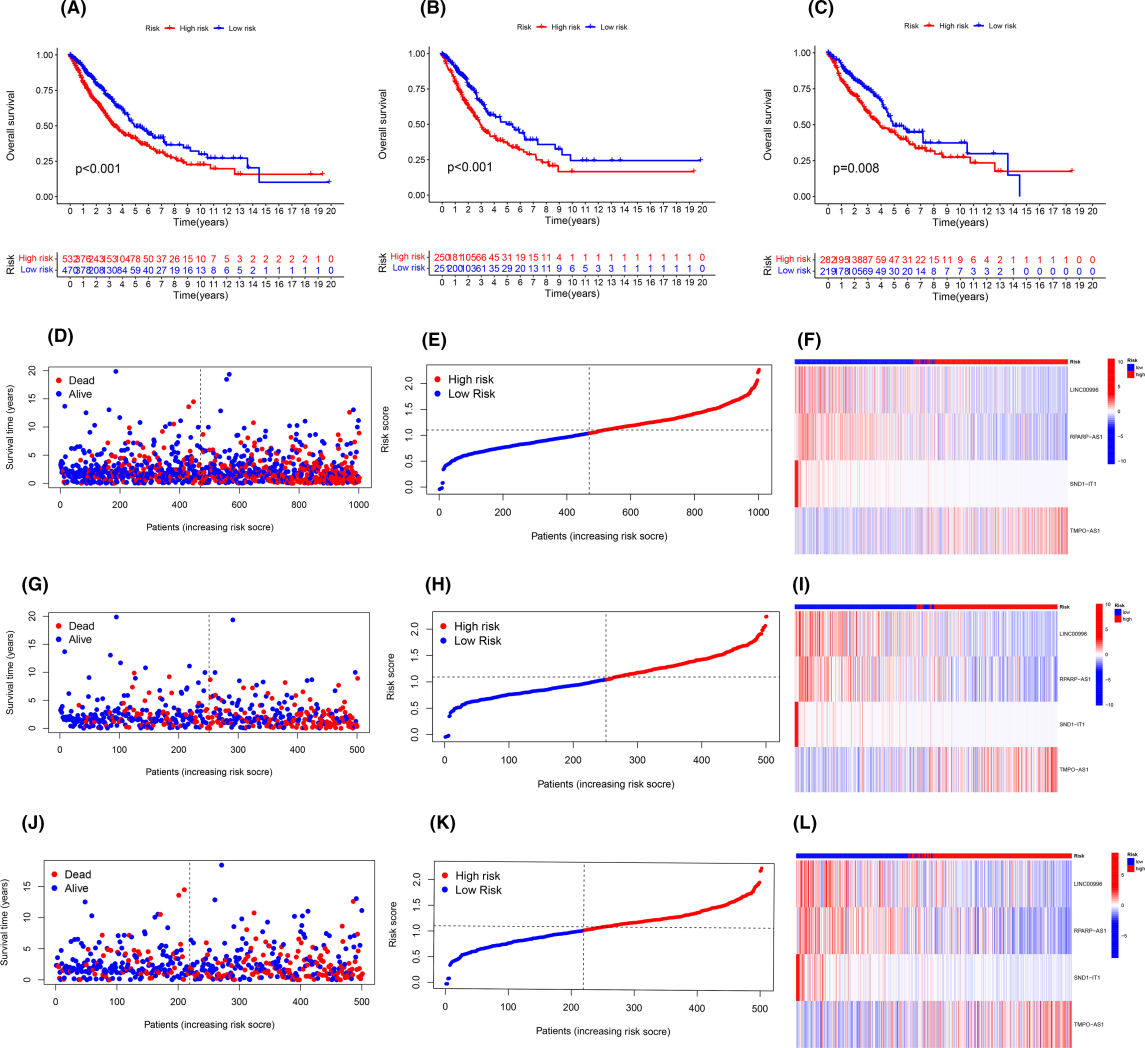
The result of survival analysis. (A) The survival curves of all samples, (B) survival curve of the training set, (C) survival curve of the validation set; (D–F) risk curve, sample distribution map, and heat map for all sample sets, (G–I) risk curve, sample distribution map, and heat map for training sets, (J–L) risk curve, sample distribution map, and heat map for validation sets.

**FIGURE 4:**
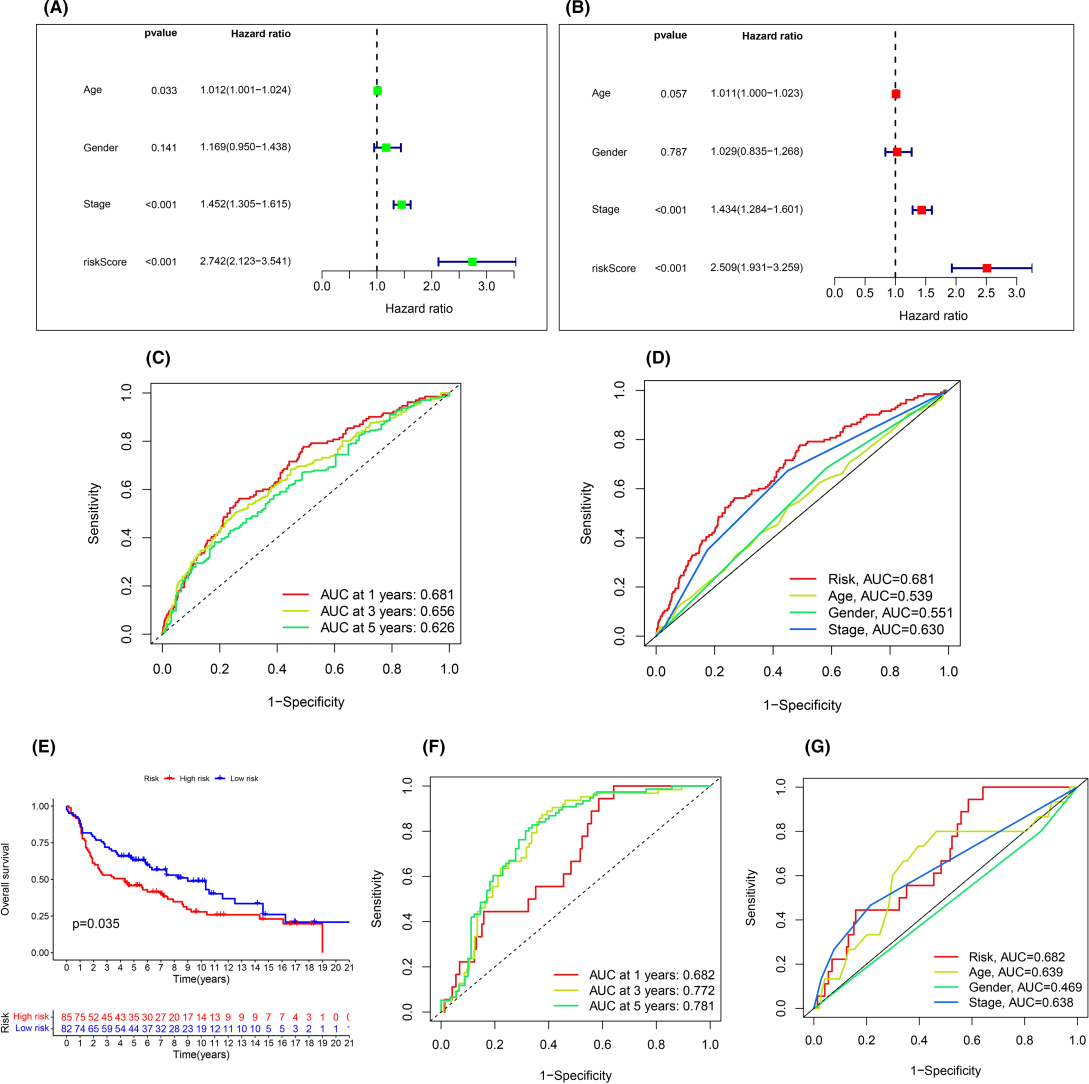
The result of independent prognostic analysis. (A) Single factor prognostic analysis. (B) Multivariate prognostic analysis results. (C) ROC curve of predicted the survival of the patients at 1, 3 and 5 years (D) ROC curve, red represents risk score, the larger the area under the curve, the greater the credibility of the results. (E) The survival curves of GEO samples, (F) ROC curve of predicted the survival of the patients at 1, 3 and 5 years, (G) ROC curve of risk values and age, gender, different staging of GEO samples.

By survival analysis, it can be seen that in all samples, training set and validation set, the survival rate of the low‐risk group is higher than that of the high‐risk group with the extension of time, and the results are displayed in Figure 3A–C. We further visualized each sample by risk curves, through which it can be seen that with the extension of time, patient deaths in the high‐risk group were higher than those in the low‐risk group, and the expression of TMPO‐AS1 was increased in the high‐risk group. LINC00996, RPARP‐AS1 and SND1‐IT1 were highly expressed in the low‐risk group, and the results are displayed in Figure 3D–L. To further verify the accuracy of model prediction, we performed independent prognostic analysis by grouping the samples into different ages, genders, different stages and different grades, respectively, and it can be seen that the risk values of our prognostic model have good prediction results in both unifactorial and multifactorial prognostic analyses, and the *p* value is less than 0.01, and the results are shown in Figure 4A,B. To further verify the accuracy of model prediction, we tested the accuracy of our model between different subgroups by grouping the samples and testing the accuracy of our model by ROC curve for different age, gender and different stages. The results are shown in Figure 4C. We further demonstrated the predictive accuracy of our prediction model at survival times of 1, 3 and 5 years through ROC curves, where the area under the ROC curve was (AUC at 1 year: 0.681; AUC at 3 year: 0.656; AUC at 5 years: 0.626), respectively. The results are shown in Figure 4D. In addition to this, we used tumour samples from the GEO database as an external validation set to further validate the reliability of the model through survival analysis and ROC curves. The survival curve shows that the survival rate of the low‐risk group is higher than that of the high‐risk group with the extension of time, the results are shown in Figure 4E. The results of the ROC curves show that the risk values have a high degree of accuracy when compared to age, gender and different staging. We further demonstrated the predictive accuracy of our prediction model at survival times of 1, 3 and 5 years through ROC curves, where the area under the ROC curve was (AUC at 1 year: 0.682; AUC at 3 year: 0.772; AUC at 5 years: 0.781). The results are shown in Figure 4F,G.

### 3.5 Column line graph to predict the survival of the sample

**FIGURE 5:**
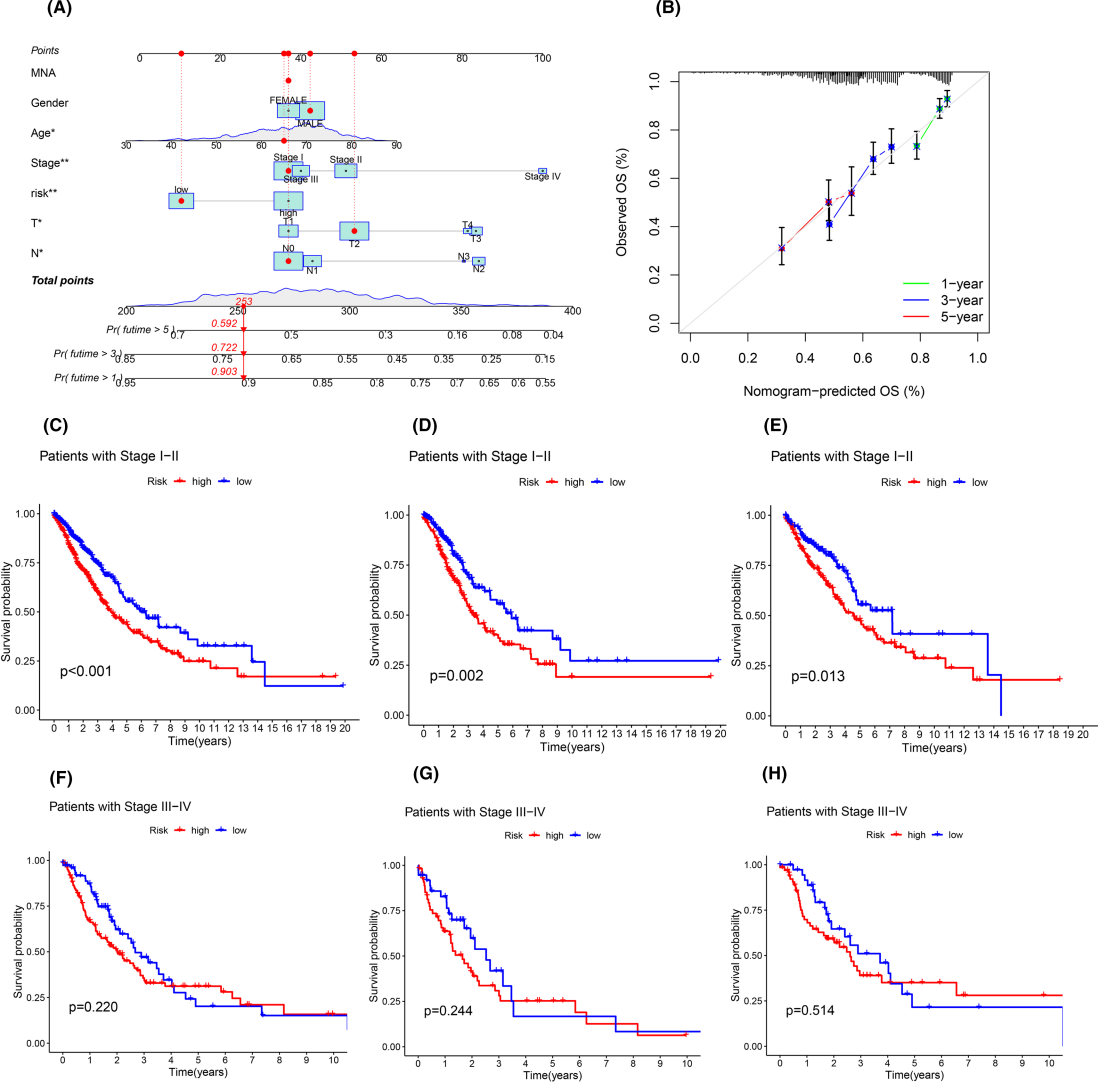
The result of column line graph to predict the survival of the sample. (A) column line graph to predict the survival of the sample, predict the 1‐, 3‐ and 5‐year survival rates of patients through scoring, using the correction curve of the (B) column chart. (C) Survival curves of early patients in all samples, (D) survival curves of early patients in train set, (E) survival curves of early patients in test set, (F) survival curves of late patients in all samples, (G) survival curves of late patients in train set, (H) survival curves of late patients in test set, red represents high risk, and blue represents low risk.

We scored the patients by age, gender, staging, T, N, M staging and risk value, and predicted the survival of the patients at 1, 3 and 5 years by the combined scoring and corrected by the calibration curve, and the results are shown in Figure 5A,B.

### 3.6 Survival analysis by clinical grouping

Patients were categorized into early‐stage patients (Stage I–II) and late‐stage patients (III–IV) by clinical staging, and the survival curves showed that there was a significant difference in survival between the high‐ and low‐risk groups in the early‐stage patients. In late‐stage patients, survival did not differ significantly between high‐ and low‐risk groups. The same results are found in all sample sets, the training set and the testing set. The results are shown in Figure 5C–H.

### 3.7 Principal component analysis

**FIGURE 6:**
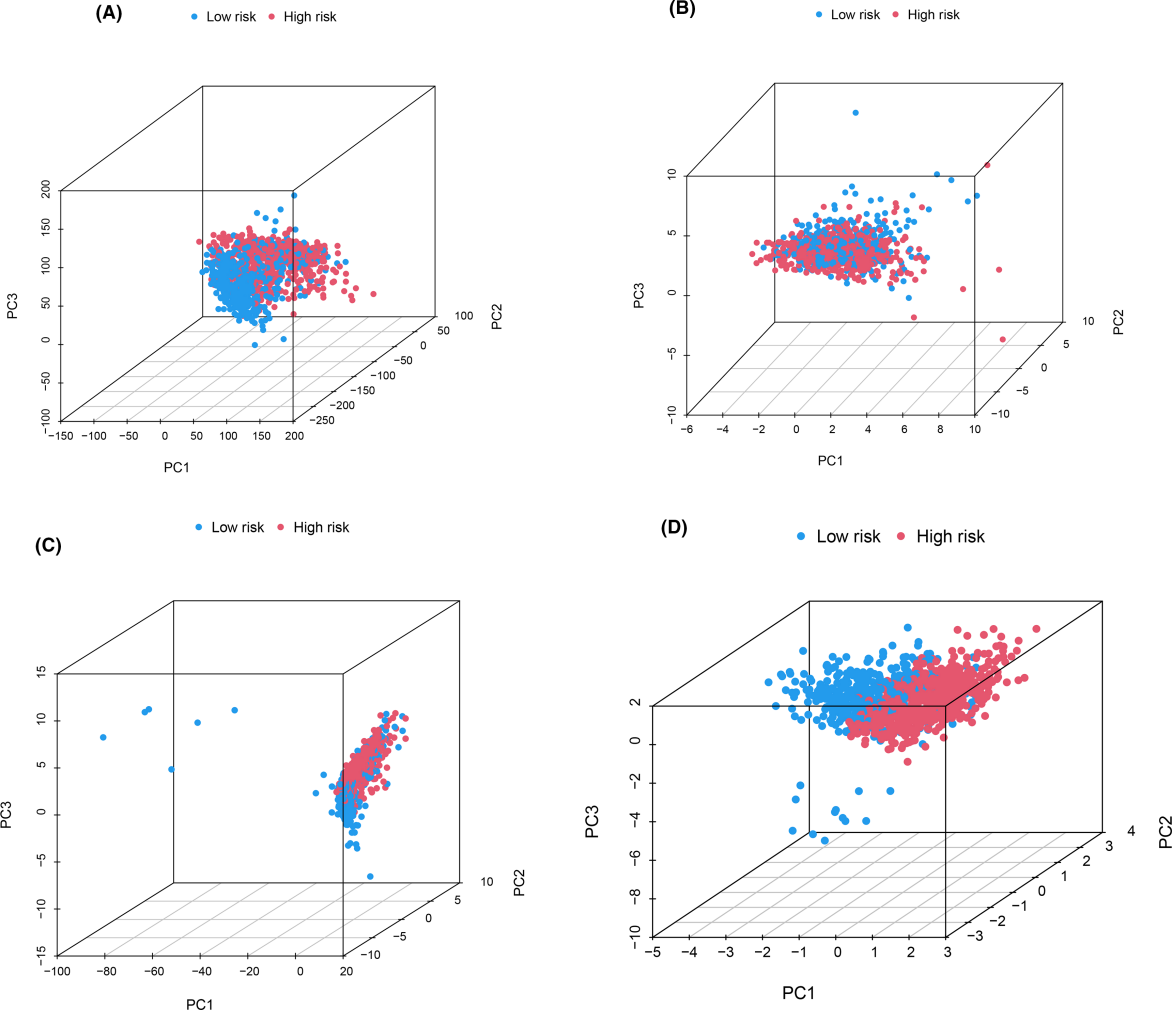
The result of PCA analysis. (A) All genes, (B) cuproptosis‐related genes, (C) cuproptosis‐related lncRNAs, (D) LncRNAs involved in model construction. It can be seen that the lncRNAs involved in model have the most significant effect in dividing high‐ and low‐risk groups.

The PCA shows whether the constructed model genes can well distinguish the samples between high and low groups among all genes, cuproptosis‐related genes, cuproptosis‐related lncRNAs, and lncRNAs involved in model construction. The results are shown in Figure 6A–D.

### 3.8 Analysis of immune‐related functions

**FIGURE 7:**
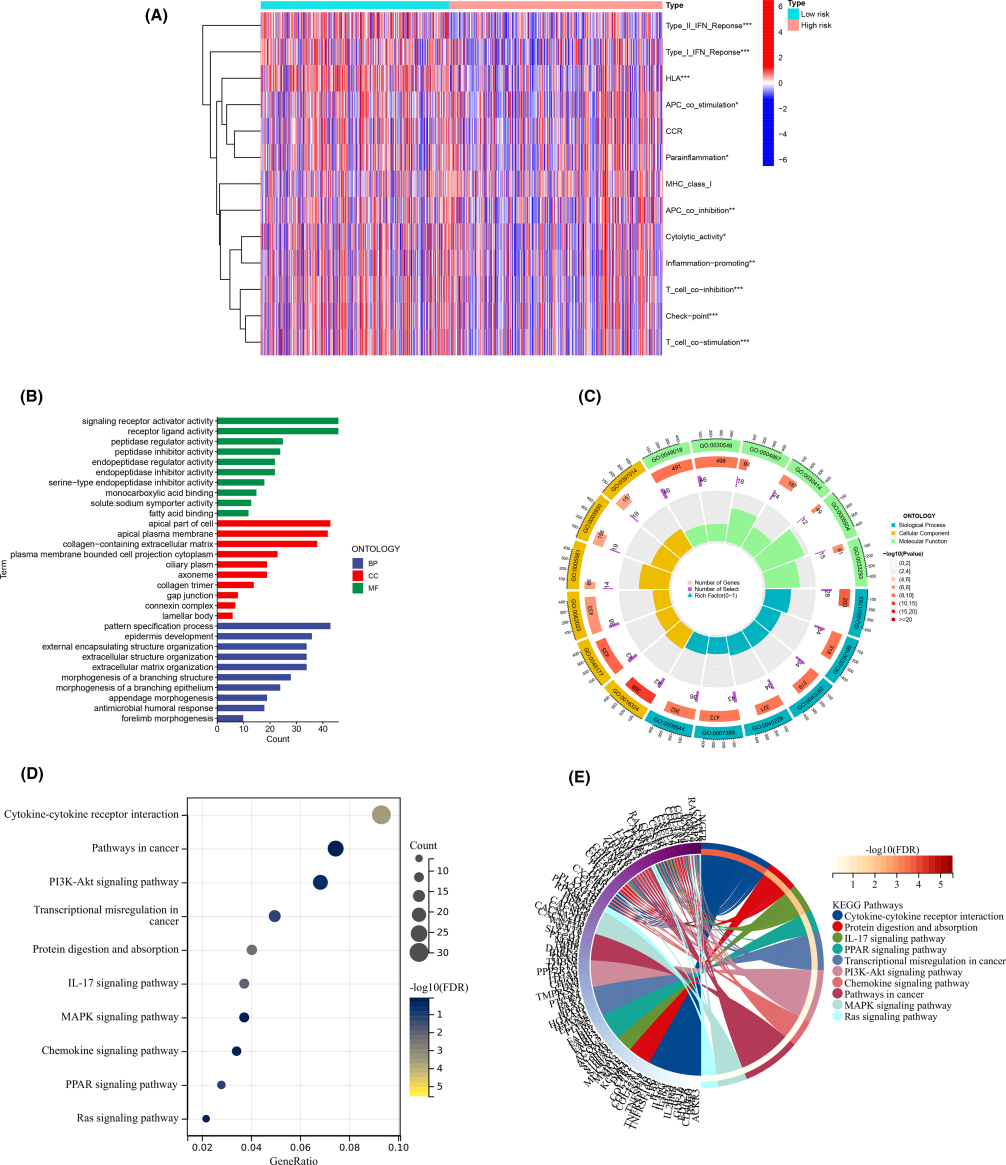
The result of Analysis of immune‐related functions and Functional enrichment analysis. (A) The result of Analysis of immune‐related functions, *p < 0.05, **p < 0.01, ***p < 0.001. (B, C) The result of GO enrichment analysis, from outside to inside, the first circle represents the ID of the GO, the second circle represents the number of genes on each GO, the colour of the second circle represents the significance of the enrichment, the redder the colour means the more significant the enrichment, the third circle represents the number of co‐expressed genes, and the fourth circle represents the heat ratio of genes. (D, E) The result of KEGG enrichment analysis, the colour of the bar graph represents the *p*‐value, the colour change from light to dark means that the *p*‐value becomes larger gradually, and the size of the endpoints represents the number of genes enriched in the pathway, the larger the endpoints the greater the number of enriched genes.

By immune‐related function analysis, it can be seen that the immune‐related functions with differences between high‐ and low‐risk groups are (Type_II_IFN_Reponse, Type_I_IFN_Reponse, HLA, T_cell_co‐inhibition, Check‐point, T_cell_co‐stimulation), and the results are shown in Figure 7A.

### 3.9 Functional enrichment analysis

By functional enrichment analysis, the results of GO enrichment analysis showed that the pathways that were more significantly enriched in the high‐risk group were (BP: forelimb morphogenesis, antimicrobial humoral response, appendage morphogenesis, morphogenesis of a branching epithelium, morphogenesis of a branching structure, extracellular matrix (ECM) organization, extracellular structure organization, external encapsulating structure organization, epidermis development, pattern specification process; CC: lamellar body, connexin complex, gap junction, collagen trimer, axoneme, ciliary plasm, plasma membrane bounded cell projection cytoplasm, collagen‐containing ECM, apical plasma membrane, apical part of cell; MF: fatty acid binding, solute: sodium symporter activity, monocarboxylic acid binding, serine‐type endopeptidase inhibitor activity, endopeptidase inhibitor activity, endopeptidase regulator activity, peptidase inhibitor activity, peptidase regulator activity, receptor ligand activity, signalling receptor activator activity), the results are shown in Figure 7B,C. KEGG enrichment analysis showed that the pathways that were more significantly enriched in the high‐risk group were (cytokine–cytokine receptor interaction, pathways in cancer, PI3K‐Akt signalling pathway, transcriptional misregulation in cancer, protein digestion and absorption, IL‐17 signalling pathway, MAPK signalling pathway, chemokine signalling pathway, PPAR signalling pathway, Ras signalling pathway), The results are shown in Figure 7D,E. According to the result of the pathways that were expressed differently between the high‐ and low‐ risk groups, after setting the filtering conditions, the pathways that were more obvious in the high‐ and low‐ expression groups were used as the next step of the study.

### 3.10 Differential analysis of tumour mutation load

**FIGURE 8:**
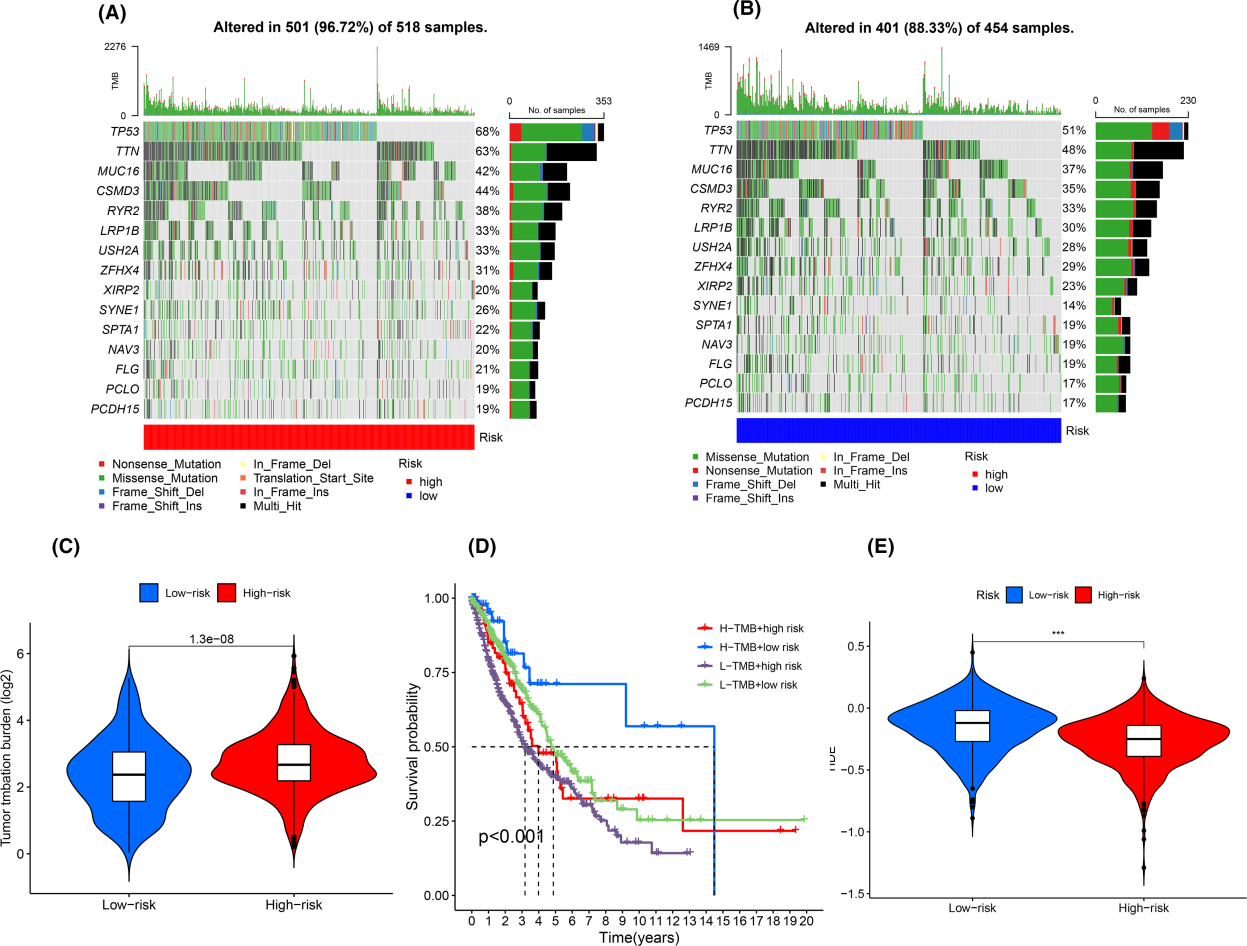
The result of tumour mutation load. (A, B) The waterfall plot of tumour mutations shows the low‐risk group in blue and the high‐risk group in red. (C) Violin diagram for differential analysis of tumour mutations. Blue represents the low‐risk group, while red represents the high‐risk group. (D) Survival analysis of tumour mutation combined with high‐ and low‐risk groups; four groups: H‐TMB + high risk, H‐TMB + low risk, L‐TMB + high risk, L‐TMB + low risk. (E) Violin diagram for tumour immune dysfunction and exclusion. Blue represents the low‐risk group, while red represents the high‐risk group.

With the tumour mutation load data of the samples in the TCGA database, combined with the risk values of the samples, we visualized the tumour mutation load through a waterfall plot, and the top three mutated genes in the high‐risk group were TP53 (68%), TTN (63%) and CSMD3 (44%), whereas the top four mutated genes in the low‐risk group were TP53 (51%), TTN (48%) and MUC16 (37%). The results are shown in Figure 8A,B. We analysed the difference in mutation loads in high‐ and low‐risk groups, and analysed the mutation differences of modelled genes between high and low risk groups, and from the results, we can see that the mutation frequency in high‐risk groups is higher than the mutation frequency in low‐risk groups. The results are shown in Figure 8C. In order to further verify the accuracy of our model, we added the risk values from the previous model to the analysis and divided them into four groups: H‐TMB + high risk, H‐TMB + low risk, L‐TMB + high risk, L‐TMB + low risk, and it can be seen in the results that there is also a difference in survival between the high‐ and low‐ risk groups among the high and low mutation groups, which further illustrates the accuracy of our model. The results are shown in Figure 8D.

### 3.11 Tumour immune dysfunction and exclusion

The analysis of immunotherapy escape, combined with the risk values of each sample in the model, shows that the high‐risk group scored lower than the low‐risk group in terms of immunotherapy escape, and had a better treatment outcome during the course of receiving immunotherapy. The results are shown in Figure 8E.

### 3.12 Drug sensitivity analysis

**FIGURE 9:**
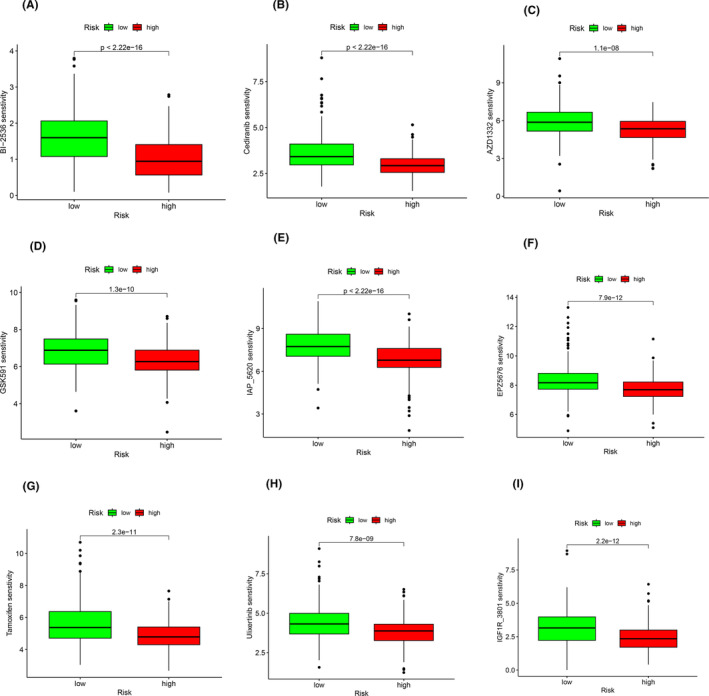
The results of drug sensitivity analysis. (A–I) The IC50 of specific drugs in different groups.

Combined with the data file of drug sensitivity in the database, we scored the drug sensitivity of each sample, and then combined with the risk value of each sample, we analysed the sensitivity of the high‐ and low‐risk groups to different drugs, through screening and eliminating the results without differences, we got the results of drug sensitivity in the high‐ and low‐risk groups have a difference in the results of the 9 drugs, the results are shown in Figure 9A–I.

### 3.13 Immune infiltration analysis

**FIGURE 10:**
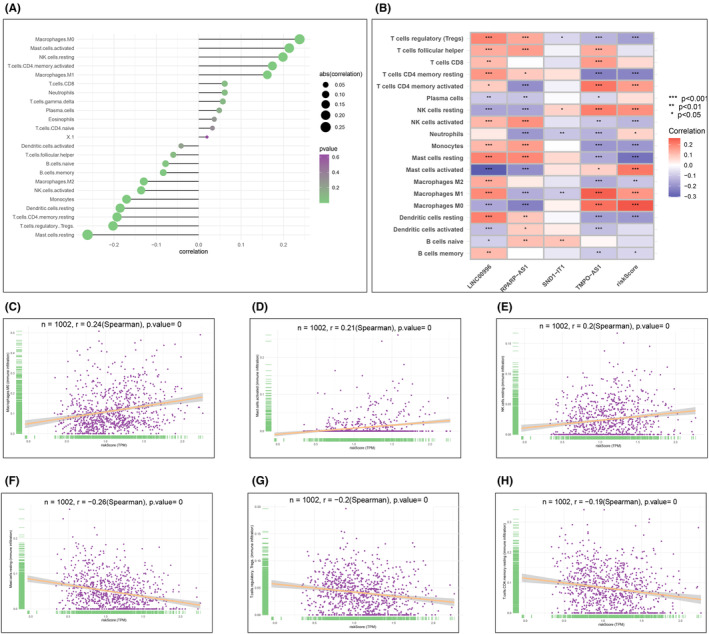
Immune microenvironment analysis. (A) Correlation between risk score and immune cells quantified by CIBERSORT algorithm; (B) correlation between model lncRNAs and immune cells quantified by CIBERSORT algorithm; (C–H) correlation between specific immune cells and risk score.

We applied the CIBERSORT algorithm to analyse the immune cell infiltration from each training set. The relative immune cell infiltration content of each sample was determined. Based on the risk values of each sample in our constructed prognostic model, we obtained the relationship between immune cells and the risk score, as well as molecules in the risk model (Figure 10A–H). The results showed that risk score was positively correlated with resting NK cells, activated CD4+ memory T cells and activated mast cells, but negatively correlated with resting CD4+ memory T cells, monocytes, resting mast cells and resting dendritic cells.

## 4 DISCUSSION

NSCLC, marked by its genetic diversity and resistance to standard therapies, presents significant challenges in the realm of patient care and prognosis.^16^ Utilizing bioinformatics has allowed for the elucidation of complex genetic alterations and molecular pathways crucial for the disease's progression and its response to treatments.^17^ This method has furthered our understanding of tumour heterogeneity and the mechanisms behind drug resistance in NSCLC, underscoring the potential of personalized medicine. Through the analysis of comprehensive genomic data and advanced analytical techniques, our research contributes to identifying predictive biomarkers and refining targeted therapies.^18^ Such efforts aim to improve patient outcomes in this difficult oncological context, moving towards more individualized treatment plans.

In our bioinformatics analysis of NSCLC samples from the TCGA database, a rigorous multi‐tiered approach was employed to ascertain the role of cuproptosis‐related lncRNAs. Through a combination of univariate, LASSO, and multivariate regression analyses, we identified 4 pivotal lncRNAs LINC00996, RPARP‐AS1, SND1‐IT1, and TMPO‐AS1. These lncRNAs were instrumental in constructing and validating a prognostic signature, highlighting their potential as biomarkers in NSCLC. Notably, our model revealed significant survival differences between high‐ and low‐risk groups of patients, underscoring the prognostic value of these lncRNAs. LINC00996, RPARP‐AS1, SND1‐IT1, and TMPO‐AS1 have emerged as key molecules in the intricate network of tumour pathophysiology, potentially influencing NSCLC progression and patient outcomes. For instance, as a mast cell marker gene, LINC00996 is hypothesized to play a critical role in regulating the occurrence and development of NSCLC and may have applications as a prognosis indicator in patients with NSCLC.^19^ RPARP‐AS1 may be involved in the progression and metastasis of colon cancer and osteosarcoma, potentially through interactions with key signalling pathways.^20^, ^21^ In addition, SND1‐IT1, notably overexpressed in exosomes secreted by gastric cancer cells, has been implicated in the malignant transformation of GES‐1 cells, primarily by upregulating SNAIL1 through an exosome‐mediated mechanism.^22^ Lastly, TMPO‐AS1, identified as a novel molecular participant in the progression of laryngeal squamous cell carcinoma (LSCC), not only contributes to the disease's pathogenic pathways but also holds potential as a viable prognostic biomarker, offering insights into the future direction of LSCC diagnostics and therapy.^23^ The collective insights from these lncRNAs provide a more nuanced understanding of the molecular landscape of NSCLC, particularly in the context of cuproptosis.

Importantly, our analysis revealed significant enrichment in several key biological processes (BP), cellular components (CC), and molecular functions (MF), including limb morphogenesis, ECM organization, and fatty acid binding. These findings provide deeper insights into the pathogenesis of NSCLC and may guide future therapeutic strategies. Notably, the pivotal role of ECM organization in the tumour microenvironment is widely recognized. Our findings support the potential role of ECM remodelling in NSCLC, particularly in tumour invasiveness and metastasis.^24^ Alterations in gap junctions, also evident in our analysis, suggest a role in cellular communication in NSCLC.^25^ We also observed a significant enrichment in fatty acid binding activity, possibly reflecting metabolic pathway alterations in tumour cells. Echoing existing research on tumour metabolic reprogramming, our results imply that NSCLC cells might support their growth and survival by altering lipid metabolism.^26^ Lastly, our analysis reveals enriched receptor ligand activity, indicating the significance of signalling pathways in tumour biology. Further research could explore the specific roles of these pathways in NSCLC progression, offering potential targets for novel targeted therapies.^27^ Overall, our study underscores the importance of bioinformatics in understanding the molecular mechanisms of cancer and provides valuable insights for further research and treatment approaches in NSCLC. Future work will need to experimentally validate these findings and explore their potential in clinical applications.

The application of the CIBERSORT algorithm to analyse immune cell infiltration has yielded insightful correlations between immune cell composition and the risk scores derived from our prognostic signature. Notably, the positive correlation of the risk score with resting NK cells, activated CD4+ memory T cells, and activated mast cells suggests a complex interplay between these immune components and tumour progression in NSCLC. The elevated presence of resting NK cells and activated CD4+ memory T cells in high‐risk cases might indicate a state of immune evasion or adaptation by the tumour cells. NK cells, generally known for their role in cancer immunosurveillance, might be in a suppressed or ineffective state in these high‐risk cases, as suggested by their ‘resting’ status. This observation aligns with studies showing that tumour cells can modify NK cell activity to evade immune responses.^28^ Similarly, the increase in activated CD4+ memory T cells in high‐risk samples could reflect a compensatory immune response to tumour progression, or alternatively, these cells might be indicative of a tumour‐promoting inflammatory environment. The role of CD4+ T cells in cancer is complex and context‐dependent, as they can exert both tumour‐suppressing and tumour‐promoting effects.^29^ These correlations underscore the intricate relationship between tumour biology and the immune microenvironment in NSCLC. They also highlight the potential of immune cell profiles as biomarkers for prognosis and as therapeutic targets.

This study contributes significantly to the field by providing a novel understanding of the molecular dynamics in NSCLC, specifically through the lens of cuproptosis‐related lncRNAs. However, further research including more experiments is necessary to unravel the precise mechanisms through which these lncRNAs influence NSCLC progression and to explore their utility in clinical settings. On the other hand, the mechanistic basis of these immune associations warrants further investigation, and it is essential to determine whether these immune cell alterations are a cause or consequence of tumour progression. Such investigations could pave the way for more personalized and effective therapeutic strategies for NSCLC patients.

## AUTHOR CONTRIBUTIONS

**Hai Huang:** Formal analysis (equal); writing – original draft (equal). **Guoxi Chen:** Writing – original draft (equal). **Zongqi Zhang:** Writing – original draft (equal). **Gang Wu:** Project administration (equal); writing – review and editing (equal). **Zhengbin Zhang:** Conceptualization (equal); writing – review and editing (equal). **Aiping Yu:** Data curation (equal); writing – original draft (equal). **Jianjie Wang:** Investigation (equal); writing – review and editing (equal). **Chao Quan:** Methodology (equal); writing – review and editing (equal). **Yuehua Li:** Methodology (equal); writing – original draft (equal). **Meilan Zhou:** Project administration (equal); writing – review and editing (equal).

## FUNDING INFORMATION

This work was supported by Hubei Province Health and Family Planning Scientific Research Project (Grant/Award Number: WJ2023F054), Hubei Provincial Natural Science Foundation Research Project (Grant/Award Number: 2022CFB176) and Scientific Research Projects from Wuhan Municipal Health Commission (Grant/Award Number: WX23B39).

## CONFLICT OF INTEREST STATEMENT

None.

## Data Availability

All data are available from the corresponding author upon reasonable request.
